# Determination of Physicochemical, Textural, and Sensory Properties of Date-Based Sports Energy Gel

**DOI:** 10.3390/gels9060487

**Published:** 2023-06-13

**Authors:** Syahrul Anis Hazwani Mohd Baroyi, Yus Aniza Yusof, Nashratul Shera Mohamad Ghazali, Alhussein M. Al-Awaadh, Kazunori Kadota, Shuhaimi Mustafa, Hazizi Abu Saad, Nor Nadiah Abdul Karim Shah, Mohammad Fikry

**Affiliations:** 1 Laboratory of Halal Science Research, Halal Products Research Institute, Universiti Putra Malaysia, Serdang 43400, Malaysia; anis1754.ah@gmail.com (S.A.H.M.B.);; 2 Department of Process and Food Engineering, Faculty of Engineering, Universiti Putra Malaysia, Serdang 43400, Malaysia; 3 Department of Agricultural Engineering, King Saud University, P.O. Box 2460, Riyadh 11451, Saudi Arabia; 4 Department of Formulation Design and Pharmaceutical Technology, Faculty of Pharmacy, Osaka Medical and Pharmaceutical University, 4-20-1 Nasahara, Osaka 569-1094, Japan; 5 Department of Microbiology, Faculty of Biotechnology and Biomolecular Sciences, Universiti Putra Malaysia, Serdang 43400, Malaysia; 6 Department of Nutrition, Faculty of Medicine and Health Sciences, Universiti Putra Malaysia, Serdang 43400, Malaysia; 7 Department of Agricultural and Biosystems Engineering, Faculty of Agriculture, Benha University, Toukh 13736, Egypt

**Keywords:** dates, black seed, energy gels, public health, physicochemical properties

## Abstract

Applying energy supplements in gel form may circumvent gastric discomfort and thus it is a practical alternative. The main objective of this investigation was to develop date-based sports energy gels consisting of highly nutritious ingredients such as black seed (*Nigella sativa* L.) extract and honey. Three date cultivars (Sukkary, Medjool, and Safawi) were used and characterized for their physical and mechanical properties. The sports energy gels were prepared with addition of xanthan gum (0.5% *w*/*w*) as the gelling agent. The newly developed date-based sports energy gels were then analysed for proximate composition, pH level, colour, viscosity, and texture profile analysis (TPA). A sensory test was also conducted with 10 panellists who analysed the appearance, texture, odour, sweetness, and overall acceptability of the gel using a hedonic scale. The results showed that different types of date cultivars affect the physical and mechanical properties of the new developed gels. The outputs of the sensory evaluation revealed that the date-based sports energy gel prepared from Medjool received the highest mean score, followed closely by those prepared from Safawi and Sukkary, indicating that, overall, all three cultivars are acceptable to consumers, but the date-based sports energy gel prepared from Medjool is the most preferred one.

## 1. Introduction

The palm date fruit, which is widely cultivated in several countries such as Egypt, Saudi Arabia, the United Arab Emirates, Algeria, and Iraq, is regarded as a healthful dietary item. Around 57% of the palm date fruit supply in the world is produced in these countries [1]. Date palm or its scientific name, *Phoenix dactylifera*, belong to the date palm family. Ajwa, Sukkary, Saquee, Ekhlass, Barhee, Anbara, Safawi, Rothana, Rashodya, and Khedry are well-known cultivars in Saudi Arabia [2]. Dates are rich in carbohydrates, vitamins, fibres, minerals, and polyphenols, and are considered one of the most important commercial products of Saudi Arabia. Dates are well known for being highly rich sources of dietary fibre, natural antioxidative nutrients including selenium, phenolics, and carotenoids, and polyphenols that produce bioactive agents such as anticarcinogenic, antimutagenic, and anti-inflammatory ones [3]. They are also high in insoluble fibre which is important for gastrointestinal health [4]. Carbohydrates, and particularly the sugars sucrose, glucose, and fructose, make up about 70% of date fruit. After ingestion, sugars are promptly transported into the blood and quickly metabolized to produce energy for a broad range of cellular activities [5]. Palm date fruits are commonly consumed in fresh, dried, or processed forms. There are some derivative products, such as juice, jam, and pastes, which can be produced from the flesh of date fruits. Date-based sports energy gel would be an innovative and convenient way to allow consumers to consume dates whenever and wherever they choose.

Meanwhile, black seed, or its scientific name, *Nigella sativa* (family: Ranunculaceae), is commonly known as black seed, black cumin, or “*Habbatus Sauda*”. Black seed contains thymoquinone, thought to be medicinally very effective against various illnesses including different chronic illness: neurological and mental illness, cardiovascular disorders, cancer, diabetes, inflammatory conditions, and infertility as well as various infectious diseases caused by bacterial, fungal, parasitic, and viral infections [6]. Over 2000 years have passed since black seed was first used as a natural remedy. In many nations, it can also be used as a flavouring ingredient and food additive. Because it contains more than one hundred active ingredients, *Nigella sativa* oil has been proven to be highly beneficial [7]. The new date-based sports energy gels that were produced in this study, by integrating dates with black seed extract and honey, will not only be utilised as an energy booster but also as a new supplement in the pharmaceutical field that can be used by all community members at all different levels.

In recent decades, food hydrocolloids have garnered considerable attention within the realm of food science and technology research due to their significant contributions to various aspects of food, such as structure, processing, stability, flavour, nutrition, and health benefits [8]. Furthermore, they have also proven to be valuable in pharmaceutical formulations [9]. Energy supplements are commonly used to sustain the required energy levels for training, athletes, and physical activities. These supplements typically rely on carbohydrates to maintain glucose levels during physical exertion. However, there are concerns regarding the potential digestive issues and stomach discomfort associated with consuming liquid sports drinks for athletes and sports enthusiasts. Utilizing energy supplements in gel form presents a practical alternative that may help to mitigate gastric discomfort [10].

The purpose of this study is to develop a sports energy gel that is free from preservatives and utilizes dates, black seeds, and honey. The gel aims to be stable, convenient, appealing to the senses, and effective in enhancing athletic performance, catering to the demanding lifestyles of athletes and individuals who frequently travel. Moreover, the gel is intended to be highly nutritious. The development of this sports energy gel using date-based ingredients and black seeds is expected to introduce an innovative approach to product development within the sports industry of Saudi Arabia, while adhering to international quality standards. These endeavours will encourage the utilization of dates and black seeds as functional ingredients in various product formulations, thus promoting their consumption for the betterment of public health and well-being.

## 2. Results and Discussion

### 2.1. Physical Properties of Safawi, Sukkary, and Medjool Dates

The moisture content of food products is indeed a critical parameter that can affect the quality and shelf life of the product. In this particular study, Table 1 showed that the moisture content of Sukkary dates was found to be the highest, followed by Medjool and Safawi dates. The dates were categorized into three groups based on their moisture content, namely soft, semi-soft, and dry dates. As the moisture content of the three varieties of dates studied was in the range of 10 to 30% [11], they were categorized as semi-soft dates.

Apart from moisture content, another parameter that was analysed in this study was the TSS (total soluble solids) content. The TSS content of Medjool dates was found to be the highest compared to Safawi and Sukkary. TTS is a measure of the concentration of dissolved solids in a solution, and is an important indicator of the sweetness of the food product. In this case, the higher TSS content in Medjool dates suggests that they may be sweeter than Safawi and Sukkary dates.

The physical dimensions of dates are important parameters that can impact their processing and storage. Larger dates may require different processing techniques compared to smaller ones. For example, size can influence drying times or the need for cutting or pitting during processing. Understanding the physical dimensions helps in optimizing processing methods and storage practices to ensure the best quality of dates [12]. In this study, the length of Medjool dates was found to be the highest compared to other samples, with a length of 43 ± 3.4 mm. This is consistent with the findings of [13], where it was also reported that Medjool dates were longer than Sukkary dates.

In terms of width, Medjool dates had the highest value, while Sukkary dates had the highest value in thickness. The geometric mean diameter, which is an important parameter in the design of separating, harvesting, sizing, and grinding machines [14], was found to be the highest for Medjool dates, followed by Sukkary and Safawi.

The physical dimensions of dates can also be related to their fruit and flesh weights, as observed in this study where Medjool dates had the highest fruit and flesh weights when compared to Sukkary and Safawi. This finding is consistent with the study by Siddiqi et al., (2020) [15], which suggested a relationship between the dimensions of dates and their fruit and flesh weights.

The bulk density of the samples was found to be in the range of 0.38 to 0.454 g/cm^3^, with Medjool dates having the highest value. Bulk density is an important parameter in the design of equipment for handling and processing dates, as it affects the efficiency of the equipment.

Sphericity is a crucial factor when considering the design of fruit handling equipment, as it reveals the fruit’s shape, i.e., whether it is oval or cylindrical [16]. Furthermore, sphericity plays a role in the distribution of stresses during processing and packaging, potentially influencing the shelf life and structural integrity of dates. Thus, examining sphericity aids in comprehending the physical attributes of dates and their potential influence on consumer acceptance and product quality [17].

In this study, Sukkary dates had the highest sphericity value, indicating that they have a more cylindrical shape compared to Medjool and Safawi dates. However, it is worth noting that all three varieties of dates studied were found to have a cylindrical shape, as the width and thickness values were found to be similar.

### 2.2. Mechanical Properties of Different Date Cultivars

Table 2 presents the results for the different mechanical properties (hardness, adhesiveness, springiness, cohesiveness, gumminess, chewiness, and resilience) of three varieties of dates (Sukkary, Medjool, and Safawi).

Hardness is a measure of the force required to compress the date, and it is expressed in Newtons. The results indicate that Safawi dates are the hardest (1064.7 g), followed by Medjool (804.2 g), and Sukkary dates (483.3 g). The letters a, b, and c in superscripts indicate statistically significant differences among the means (*p* < 0.05). This suggests that the hardness of the three varieties is significantly different, and Safawi dates are the hardest of the three. Adhesiveness is a measure of the force required to overcome the attractive forces between the date and the surface it is in contact with. A negative value indicates that the date is adhesive. The results show that Medjool dates (−4.258 g.s) are the most adhesive, followed by Safawi (−2.497 g.s) and Sukkary (−0.596 g.s) dates. The significant difference in the adhesiveness values suggests that the three varieties differ significantly in their adhesive properties, and Medjool dates are the most adhesive of the three. Springiness is a measure of the extent to which the date returns to its original shape after deformation. The results show that the three varieties have similar springiness values, with Sukkary (0.850) and Medjool (0.843) being slightly less springy than Safawi (0.900). Cohesiveness is a measure of the internal strength of the date, and it indicates how well the date holds together when chewed. The results show that Sukkary (0.724) dates are the most cohesive, followed by Medjool (0.700) and Safawi (0.689) dates. The significant difference in cohesiveness values suggests that Sukkary dates are the most cohesive of the three. Gumminess is a measure of the energy required to masticate the date until it is ready for swallowing. The results show that Safawi (747.8) dates are the gummiest, followed by Medjool (569.6) and Sukkary (346.4) dates. The significant difference in gumminess values suggests that Safawi dates are the gummiest of the three. Chewiness is a measure of the work required to masticate the date to a state ready for swallowing. The results show that Safawi (674.5) dates are the chewiest, followed by Medjool (497.3) and Sukkary (357.6) dates. The significant difference in chewiness values suggests that Safawi dates are the chewiest of the three. Resilience is a measure of the extent to which the date recovers its original shape after being compressed. The results show that the three varieties have similar resilience values, with Sukkary (0.218), Medjool (0.198), and Safawi (0.212) having comparable values.

In general, Table 2 indicates that the three date varieties differ significantly in their properties, with Safawi dates being the hardest, gummiest, and chewiest, while Medjool dates are the most adhesive. Sukkary dates are the most cohesive but are the least gummy and chewiest of the three. These results could be comparable with the results found by Al-Awaadh, et al. [16]. The acceptable limits of the mechanical properties may differ based on regional and cultural preferences. Some varieties of dates are traditionally consumed at different stages of ripeness, which can impact their mechanical properties and consumer acceptance. Additionally, the intended use of the dates, such as direct consumption, ingredient incorporation, or industrial processing, may also influence the acceptable limits of mechanical properties.

Understanding the mechanical properties of date fruits used in gel preparation is crucial for determining suitable processing machinery, such as seed separation machines for seed removal and grinders for date grinding. This knowledge aids in making informed decisions about the appropriate equipment required for the efficient processing of dates in gel production.

### 2.3. Physicochemical Properties of Date-Based Sports Energy Gel

Table 3 shows the composition of three types of date-based sports energy gels prepared from Sukkary, Medjool, and Safawi. In terms of total ash content, the gel prepared from Medjool dates had the highest value (0.6 ± 0.00 g/100 g), followed by Safawi (0.45 ± 0.06 g/100 g) and Sukkary (0.40 ± 0.00 g/100 g). Regarding moisture content, the gel prepared from Sukkary dates had the highest value (58.33 ± 0.42 g/100 g), followed by Medjool (55.75 ± 0.59 g/100 g) and Safawi (55.85 ± 0.65 g/100 g). In terms of protein content, the gel prepared from Medjool dates had the highest value (0.80 ± 0.14 g/100 g), followed by the gels prepared from Sukkary (0.77 ± 0.05 g/100 g) and Safawi (0.63 ± 0.05 g/100 g). Date proteins are known to contain 23 types of amino acids, some of which are not present in other common fruits such as oranges, apples, and bananas [5]. Most date cultivars contain amino acids such as lysine, histidine, arginine, aspartic acid, threonine, glutamic acid, serine, proline, glycine, alanine, cystine, valine, methionine, isoleucine, leucine, tyrosine, and phenylalanine [5].

For total fat content, the gel prepared from Medjool dates had the highest value (0.20 ± 0.00 g/100 g), followed by the gels prepared from Safawi and Sukkary with similar values (0.18 ± 0.05 g/100 g and 0.16 ± 0.05 g/100 g, respectively). Although fat does not contribute significantly to the nutritional value of date flesh, it does play an essential role in protecting the product [16].

Regarding carbohydrate content, the gels prepared from Medjool and Safawi dates had similar values (42.65 ± 0.68 g/100 g and 42.9 ± 0.61 g/100 g, respectively), both being higher than the value for the gel prepared from Sukkary (40.34 ± 0.39 g/100 g). The energy content follows a similar trend as the carbohydrate content, with the gel prepared from Medjool dates having the highest value (175.5 ± 2.38 kcal/100 g), followed by the gels prepared from Safawi and Sukkary with similar values (175.5 ± 2.52 kcal/100 g and 166 ± 1.63 kcal/100 g, respectively). This is in line with previous studies, such as the work by Aljaloud et al., (2020) [18], which have shown that date fruit is a good source of carbohydrates and sugars.

In terms of pH, Sukkary dates have the highest value (5.35 ± 0.04), indicating a slightly alkaline pH, followed by the gels prepared from Medjool and Safawi with similar values (5.11 ± 0.07 and 5.10 ± 0.02, respectively), indicating a slightly acidic pH. 

Table 3 presents the results of the colour analysis of the date-based sports energy gels prepared from three varieties of dates: Sukkary, Safawi, and Medjool. The colour analysis is based on the CIELAB colour space, which has three components: L* (lightness), a* (red–green axis), and b* (yellow–blue axis).

The results show that the date-based sports energy gels prepared from Sukkary and Medjool dates have similar L* values (32.42 and 31.07, respectively), while the gel prepared from Safawi dates has the lowest L* value (30.38). This suggests that Safawi dates are darker than the other two varieties.

In terms of the a* component, the gel prepared from Medjool dates has the highest value (5.05), followed by the gel prepared from Sukkary (4.30), and the gel prepared from Safawi (3.72) dates. The significant difference in the a* values suggests that Medjool dates have a more reddish hue than the other two varieties.

For the b* component, the date-based sports energy gel prepared from Sukkary dates has the highest value (6.72), followed by the gel prepared from Medjool (6.93), and the gel prepared from Safawi (6.29) dates. The significant difference in the b* values suggests that Sukkary dates have a more yellowish hue than the other two varieties.

Generally, the colour analysis results show that the date-based sports energy gels prepared from the three date varieties have distinct colour profiles. The gel prepared from Safawi dates was darker, while the gel prepared from Medjool dates had a more reddish hue. The gel prepared from Sukkary dates had a more yellowish hue than the other two varieties. These colour differences can be attributed to variations in the amount and type of pigments present in each variety [16]. The colour information provided in the table can be useful for quality control and product development purposes in the date industry.

**Figure 1:**
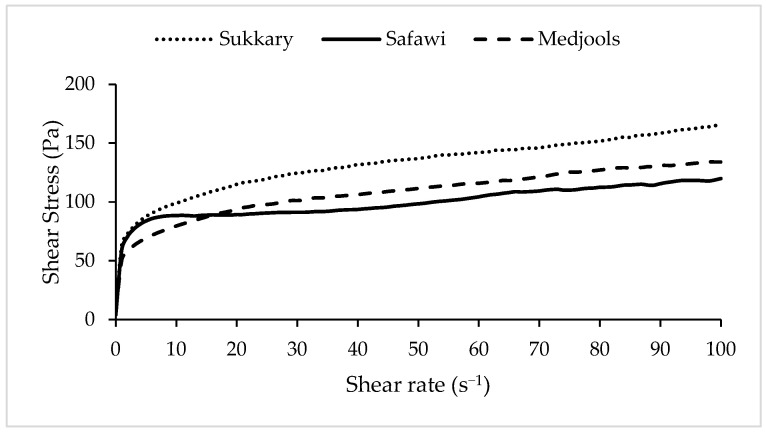
Flow curve of date-based sports energy gel.

The viscosity results showed that the date-based sports energy gels made from Sukkary had the highest viscosity of 11,417 ± 587 mPa·s, followed by Medjool and Safawi date-based sports energy gels which had values of 10,539 ± 1323 mPa·s and 8371 ± 972 mPa·s, respectively. These results were found to be in coherence with the mechanical properties for each date cultivar. The lower the hardness, chewiness, and gumminess of the dates, the more viscous the gel produced will be. The viscosity of the gels was also affected by the type and concentration of the gelling agent used in the gel production. The viscosities in the present study were found to be higher than in gels studied by Lestari, et al., (2021) [19] and are in the range of viscous liquids reported in Rao (1977) [20], as the authors used only 0.1% of xanthan gum compared to 0.5% of xanthan gum which was used in this new developed date-based sports energy gel.Based on the flow curve of the date-based sports energy gels presented in Figure 1, it is shown that this newly developed sports energy gel behaves rheologically as a non-Newtonian fluid with shear thinning properties, where its viscosity decreases with increasing shear rate, leading to a perception of it being easy to swallow [21] which is a pertinent attribute for sports energy gels.

The results of the texture profile analysis (TPA) of the date-based sports energy gel provide important information on the physical properties of the gel that can influence its overall quality and acceptability. Hardness and gumminess are the most pertinent sensory attributes that can affect the texture of the gel, and, in turn, its palatability. 

Table 3 shows the mean values of different texture properties for date-based energy gels prepared from three types of dates: Sukkary, Medjool, and Safawi. In terms of gumminess, there were no significant differences between the three types of dates, with values ranging from 11.23 to 13.17. Hardness values were also similar for the date-based energy gels prepared from the three types of dates, with the date-based energy gel prepared from Sukkary being slightly harder (1.27 ± 0.47 g) than those prepared from Medjool (1.14 ± 0.57 g) and Safawi (1.11 ± 0.59 g). These results could be comparable with those found for date paste by Alhamdan, et al. [22]. The authors found that the maturity stage of the dates is one of the reasons that might affect the textural properties of date paste, especially regarding hardness. The different maturity stages of dates display variations in moisture value, sugars, pectin, and cellulose materials. All the dates used in this study were at the Tamer (fully ripped) maturity stage, which was represented in the obtained textural analysis as no significant changes in hardness were found for any of the date cultivars [22].

In general, softer gels are preferred by consumers, as indicated by the lower range of hardness values found in the study. Meanwhile, gumminess is an attribute that describes the force required to chew the gel, and higher values indicate a rubberier texture.

### 2.4. Sensory Properties of the Date-Based Sports Energy Gel

**Figure 2:**
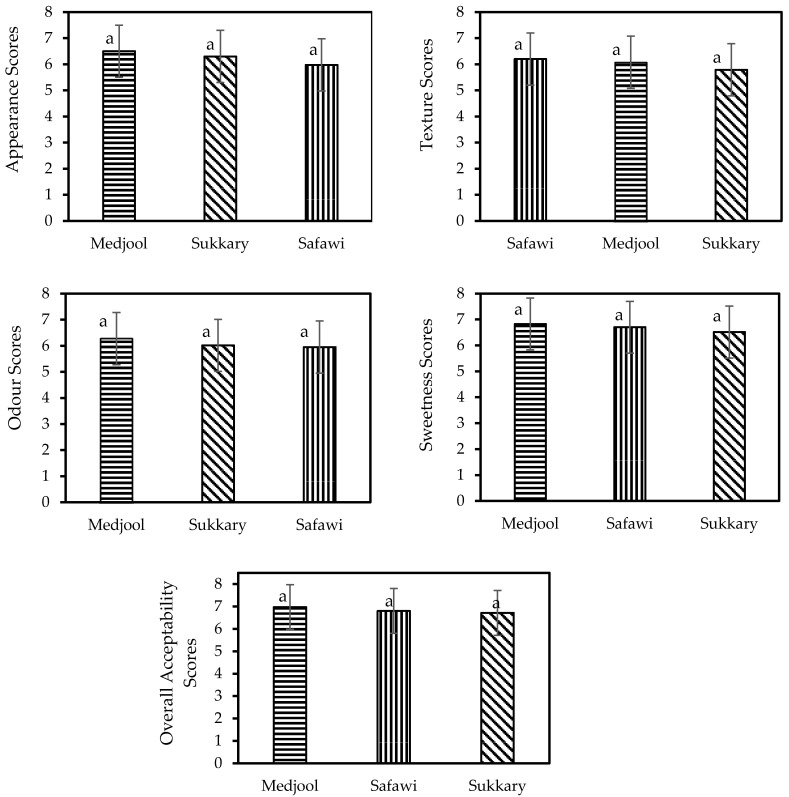
Sensory attributes of the date-based sports energy gel as affected by the date cultivar. Note: Supercript letter (^a^) indicating that there is no statistical differences were obtained between date cultivars.

The sensory properties of the date-based sports energy gel refer to its appearance, texture, odour, sweetness, and overall acceptability, as perceived by consumers (*n* = 10). Evaluating the sensory properties of food is important for understanding how it is perceived and whether it is likely to be well-received by consumers. A sensory evaluation of the date-based sports energy gel was performed to assess consumer perception and acceptability. Figure 2 presents the mean and standard deviation for four properties (appearance, texture, odour, sweetness), and the overall acceptability of the date-based sports energy gels prepared from three cultivars of dates (Medjool, Sukkary, Safawi). From Figure 1, it can be observed that the date-based sports energy gel prepared from the Medjool cultivar received the highest mean score for appearance, odour, sweetness, and overall acceptability, indicating that it is perceived as the most visually appealing, aromatic, sweet, and overall preferable cultivar among the three.

The date-based sports energy gel prepared from Safawi and Medjool cultivars received the highest mean score for texture, with that prepared from Safawi having a slightly higher score. This suggests that these two cultivars have a firmer texture than the gel prepared from Sukkary.

The date-based sports energy gel prepared from the Sukkary cultivar received the lowest mean score for appearance, texture, and odour, indicating that it is perceived as the least visually appealing, has the softest texture, and is the least aromatic among the three cultivars.

For overall acceptability, the date-based sports energy gel prepared from Medjool received the highest mean score, followed closely by those prepared from Safawi and Sukkary, indicating that, overall, all three cultivars are acceptable to consumers, but the date-based sports energy gel prepared from Medjool is the most preferred one.

However, the analysis of variance displayed that there are no significant differences in the appearance, texture, odour, sweetness, and overall acceptability values of gels prepared from Sukkary, Medjool, and Safawi. These results could be comparable with those found for date jam by Alqahtani, et al. [23].

## 3. Conclusions

This study investigated the impact of date cultivar on the physical, mechanical, and sensory properties of the date-based sports energy gel. In summary, date-based sports energy gel is a rich source of carbohydrates, and it provides essential amino acids required for the metabolic functioning of the human body. Although the fat content is relatively low, it plays a crucial role in protecting the product. In terms of gumminess, there were no significant differences between the three types of dates. Hardness values were also similar, with the date-based energy gel prepared from Sukkary being slightly harder than those prepared from Medjool and Safawi. In addition, the outputs of the sensory evaluation revealed that the date-based sports energy gel prepared from Medjool received the highest mean score, followed closely by those prepared from Safawi and Sukkary, indicating that, overall, all three cultivars are acceptable to consumers, but the date-based sports energy gel prepared from Medjool is the most preferred one. Overall, the outcomes of this work provide useful information for the production and quality control of date-based sports energy gels.

## 4. Materials and Methods

### 4.1. Materials

Three types of date cultivars were used in this study, which were Safawi, Sukkary, and Medjool. The samples were bought at Makkah & Almadinah Market, Wholesale Yemen Trading Sdn. Bhd., Bangi, Selangor, Malaysia. Ten dates were randomly chosen from a total of one hundred dates for each cultivar to be used in determination of physical properties of dates. Three hundred grams of date was purchased from the same source and used in sports energy gel preparation. Black seed extract (Sawda, Serdang, Malaysia) was bought from the local market, and food-grade xanthan gum (NineLife, Lewes, DE, USA) was used as the gelling agent and was bought from a local bakery shop. Honeybee honey (AlShifa, Dammam, Saudi Arabia) was used in honey formulation. 

### 4.2. Physical Properties of Dates

The moisture content of the dates was assessed following the recommended method outlined by AOAC (2005) [24]. Approximately 5 g of each type of date flesh was weighed and then dried in an oven at 105 °C for 24 h. The samples were periodically weighed until a constant weight was achieved. Equation (1) was utilized to calculate the moisture content (%) of the dates:

𝑀𝑜𝑖𝑠𝑡𝑢𝑟𝑒 𝑐𝑜𝑛𝑡𝑒𝑛𝑡 (%) = (𝑀1 − 𝑀2) 𝑀1 × 100(1)

Here, *M*1 represents the initial weight of the samples, and *M*2 represents the final weight of the samples after oven-drying.

To determine the total soluble solids content (TSS) of the dates, the chopped dates were mixed with distilled water in a 1:1 ratio and triturated in a ceramic mortar. The resulting solution was filtered, and the TSS content was measured using a Hand-Held Refractometer (ATAGO Co. Ltd., Tokyo, Japan) by placing a drop of the solution onto the refractometer.

The dimensions of the fruit, including length (*L*), width (*W*), and thickness (*T*), were measured using vernier callipers. The geometric mean diameter (*Dg*) and sphericity index (*Ø*) were calculated using Equations (2) and (3), respectively [25,26]:

𝐷𝑔 = (𝐿𝑊𝑇)13(2)

∅ = 𝐷𝑔𝐿(3)

The individual fruits were weighed using an analytical weight balance. Subsequently, the pits were removed, and the weights of the fruit flesh and pits were recorded. The fruit density was determined using Equation (4). The fruits were weighed in the air and then immersed in a beaker filled with water [27]:

*ρ*𝑓 = s𝑀𝑎𝑀𝑎 − 𝑀𝑤*ρ*𝑤 (4)

Here, *ρ*𝑓 and *ρ*𝑤 represent the densities of the fruit and water (g/m^3^), respectively, and *Ma* and *Mw* are the masses of the date in the air and water, respectively. The bulk density was determined as the ratio of the weight of the fruits to the volume occupied by the same fruits [28].

### 4.3. Texture Profile Analysis (TPA) of Dates

TPA tests were performed by employing a texture analyser (TA-HD plus, Stable Micro Systems, Godalming, UK) equipped with a 75 mm diameter compression disk (P75) that was used to apply two-cycle compression force on the whole date fruit where the force-time curve was measured and analysed using the software Texture Expert for Window 1.20 (Stable Micro Systems) [16]. The experiment was conducted at a crosshead speed of 0.5 mm/s and deformed to a 5 mm depth. After the first cycle, which also imitated the first bite, the plunger was reversed upward at 1.5 mm/s and the cycle was repeated for a second cycle to imitate the second bite.

### 4.4. Preparation of Sports Energy Gels

The ingredients used for the newly developed sports energy gels were dates (Safawi, Sukkary, and Medjool), black seed extract (*Nigella sativa* L.), honey, and xanthan gum as the gelling agent. Black seed extract was bought from the local company Sawda, and a food-grade gelling agent was used in this study. The composition of the sports energy gel was as follows: 75% date paste, 2.5% black seed extract, 0.5% xanthan gum, and 22% honey. All percentages are based on weight to weight (%*w*/*w*) compositions for all ingredients. The date paste was prepared by grinding pitted dates and water with a 1:2 ratio. The finest texture of date paste needed to be obtained prior to the gel’s preparation. The date-based sports energy gel was then produced by mixing all the other ingredients into the date paste without using heat, as heating black seed extract and honey should be avoided to retain their nutritional values. Then, the mixture was homogenized for 30 min and placed in the chiller for 24 h for a complete gelation process. 

### 4.5. Properties of the Developed Product

#### 4.5.1. Proximate Analysis, pH, and Colour Analysis

All properties of the proximate composition of the energy gels were determined following the standard method of AOAC with method numbers of 923.03, 950.46, 981.10, and 991.36 for total ash, moisture, protein and total fat, respectively [29]. Carbohydrate and energy were obtained by difference and calculation, respectively. pH was measured by using a pH meter (Model 320, Metler-Toledo Ltd., Essex, UK) where samples were diluted to 1:10 ratio and mixed for 1 min prior to measurements (Akesowan & Choonhahirun, 2019). A colorimeter (WR-10, FRU, Shenzen, China) was employed to measure the Hunter colour scales, L* (0 = black, 100 = white), a* (+ = red, − = green), and b* (+ = yellow and − =blue) of the samples. 

#### 4.5.2. Texture Profile Analysis (TPA)

Texture profile analysis (TPA) was carried out following the method by da Silva Costa et al., (2020) [30] where a texture analyser (TA-HD plus, Stable Micro Systems, Surrey, UK) was employed to run TPA tests, and was equipped with a 12.7 mm diameter probe (Delrin P/0.5R) and a 50 kg load cell. The experimental conditions were fixed at 2.0 mm s^−1^ for all pre-test speeds, test-speeds and post-test speeds, and involved a 50% compression with 4.793 g of trigger force and a data acquisition rate of 100 points per second. All measurements were conducted in triplicate, and the results of hardness and gumminess were automatically calculated and collected by using the software Texture Expert for Window 1.20 (Stable Micro Systems). 

#### 4.5.3. Viscosity

A rheometer (Physica MCR 301, Anton Paar Co., Ltd., Graz, Austria) was employed following the method by Xi et al., (2019) [31] to characterize the rheological attributes of the sports energy gels using a 50 mm diameter parallel plate measurement cell (PP50) where the gap was set at 1.0 mm. The analysis was conducted at 25 °C and maintained using a water circulation system (Viscotherm VT2, Paar Physica, Anton Paar Co., Ltd., Graz, Austria). Gels were placed in room temperature conditions 1 h prior to analysis. The shear rate was increased from 0.01 to 100 s^−1^ to determine the apparent shear rate viscosity, where the final value was obtained by the average of 100 points of intervals.

### 4.6. Sensory Analysis

A nine-point hedonic scale (1 = extremely dislike to 9 = extremely like) [32] was used in the sensory analysis. It involved 10 untrained panellists who were randomly selected and who received a brief introduction about nine-point hedonic scale measurements prior to the analysis. The panellists were asked about a few properties including appearance, odour, texture, sweetness, and overall acceptability. All sports energy gels used in this test were freshly prepared and served in a testing cup, and were coded randomly. All panellists were instructed to clean their palate with water in-between each sample tasting during the evaluation.

### 4.7. Statistical Analysis

Minitab statistical software (version 17) was used in data analysis. To determine the statistical significance difference between the terms, one-way analysis of variance (ANOVA) was used with post hoc Tukey’s test at *p* < 0.05.

## Figures and Tables

**Table 1:** Physical properties of Safawi, Sukkary, and Medjool dates.

Variables	Safawi	Sukkary	Medjool
Moisture content (%)	23.8 ^c^ ± 0.2	28.8 ^a^ ± 0.6	25.2 ^b^ ± 0.4
Total soluble solids (ºBrix)	36.3 ^b^ ± 0.1	35.1 ^c^ ± 0.1	37.9 ^a^ ± 0.1
Length (mm)	38.3 ^b^ ± 2.2	33.5 ^c^ ± 1.5	43.0 ^a^ ± 3.4
Width (mm)	21.1 ^b^ ± 0.9	25.9 ^a^ ± 1.8	26 ^a^ ± 2.6
Thickness (mm)	17.9 ^b^ ± 1.8	23.3 ^a^ ± 2.7	22.3 ^a^ ± 2
Geometric mean diameter (mm)	24.4 ^a^ ± 1.1	27.2 ^a^ ± 1.4	29.2 ^a^ ± 1.8
Fruit weight (g)	8.8 ^c^ ± 1.7	12.2 ^b^ ± 1.5	17 ^a^ ± 1.7
Flesh weight (g)	7.9 ^c^ ± 1.6	10.9 ^b^ ± 1.3	15.8 ^a^ ± 1.7
Pit weight (g)	0.9 ^b^ ± 0.1	1.2 ^a^ ± 0.3	1.2 ^a^ ± 0.1
Bulk density (g/cm^3^)	0.388 ^b^ ± 0.021	0.380 ^b^ ± 0.001	0.454 ^a^ ± 0.02
Sphericity	0.64 ^a^ ± 0.03	0.81 ^a^ ± 0.05	0.68 ^a^ ± 0.05

Note: The differences are notated with superscript letter (^a,b,c^), indicating the significant differences (*p* < 0.05) between date cultivars.

**Table 2:** Mechanical properties of Safawi, Sukkary, and Medjool dates.

Properties	Sukkary	Medjool	Safawi
Hardness (g)	483.3 ^c^ ± 93.4	804.2 ^b^ ± 84.4	1064.7 ^a^ ± 68.3
Adhesiveness (g.s)	−0.596 ^a^ ± 0.24	−4.258 ^b^ ± 0.65	−2.497 ^c^ ± 0.143
Springiness	0.850 ^a^ ± 0.06	0.843 ^a^ ± 0.05	0.900 ^a^ ± 0.003
Cohesiveness	0.724 ^a^ ± 0.055	0.700 ^a^ ± 0.016	0.689 ^a^ ± 0.027
Gumminess	346.4 ^c^ ± 41.6	569.6 ^b^ ± 56.7	747.8 ^a^ ± 64.5
Chewiness	357.6 ^b^ ± 118.7	497.3 ^ab^ ± 52.2	674.5 ^a^ ± 58.2
Resilience	0.218 ^a^ ± 0.034	0.198 ^a^ ± 0.011	0.212 ^a^ ± 0.015

Note: The differences are notated with superscript letter (^a,b,c^), indicating the significant differences (*p* < 0.05) between date cultivars.

**Table 3:** Physicochemical properties of sports energy gel as affected by the date cultivar.

Composition	Sukkary	Medjool	Safawi
Total Ash (g/100 g)	0.40 ^c^ ± 0.00	0.6 ^a^ ± 0.00	0.45 ^b^ ± 0.06
Moisture (g/100 g)	58.33 ^a^ ± 0.42	55.75 ^b^ ± 0.59	55.85 ^b^ ± 0.65
Protein (g/100 g)	0.77 ^a^ ± 0.05	0.80 ^a^ ± 0.14	0.63 ^b^ ± 0.05
Total Fat (g/100 g)	0.16 ^a^ ± 0.05	0.20 ^a^ ± 0.00	0.18 ^a^ ± 0.05
Carbohydrate (g/100 g)	40.34 ^b^ ± 0.39	42.65 ^a^ ± 0.68	42.9 ^a^ ± 0.61
Energy (kcal/100 g)	166 ^b^ ± 1.63	175.5 ^a^ ± 2.38	175.5 ^a^ ± 2.52
pH	5.35 ^a^ ± 0.04	5.11 ^b^ ± 0.07	5.10 ^b^ ± 0.02
L*	32.42 ^a^ ± 0.19	31.07 ^b^ ± 0.41	30.38 ^c^ ± 0.08
a*	4.30 ^b^ ± 0.15	5.05 ^a^ ± 0.34	3.72 ^c^ ± 0.07
b*	6.72 ^a^ ± 0.41	6.93 ^a^ ± 0.63	6.29 ^a^ ± 0.15
Viscosity (mPa·s)	11,417 ^a^ ± 587	10,539 ^a^ ± 1323	8371 ^a^ ± 972
Hardness (g)	1.27 ^a^ ± 0.47	1.14 ^a^ ± 0.57	1.11 ^a^ ± 0.59
Gumminess	11.23 ^a^ ± 5.33	12.45 ^a^ ± 6.37	13.17 ^a^ ± 7.19

Note: The differences are notated with superscript letter (^a,b,c^), indicating the significant differences (*p* < 0.05) between date cultivars. L* (lightness), a* (red–green axis), and b* (yellow–blue axis).

## Data Availability

Not applicable.
